# A guidewire-parallel laser fiber preloading technique for peroral pancreatoscopy-guided pancreatic duct stone lithotripsy

**DOI:** 10.1055/a-2849-5990

**Published:** 2026-04-24

**Authors:** Tadashi Toyohara, Michihiro Yoshida, Yasuki Hori, Akihisa Kato, Kenichi Haneda, Akihisa Adachi, Yusuke Kito

**Affiliations:** 1 Department of Gastroenterology and Metabolism 38386 Nagoya City University Graduate School of Medical SciencesNagoyaJapan

Recent advances in endoscopic devices have increased the use of laser lithotripsy for pancreatic duct stones 1 2 3 . However, safe and smooth laser fiber insertion remains challenging because of the limited working space within the peroral pancreatoscopy (POPS) and the rigidity of the laser fiber, which makes it difficult to pass across the sharp angulation imposed by the mother duodenoscope and POPS tip. In addition, advancing the POPS into the pancreatic duct without guidewire assistance may be technically demanding. We present a guidewire-parallel laser fiber preloading technique that facilitates laser fiber advancement during pancreatic duct stone lithotripsy.

**Fig. 1:**
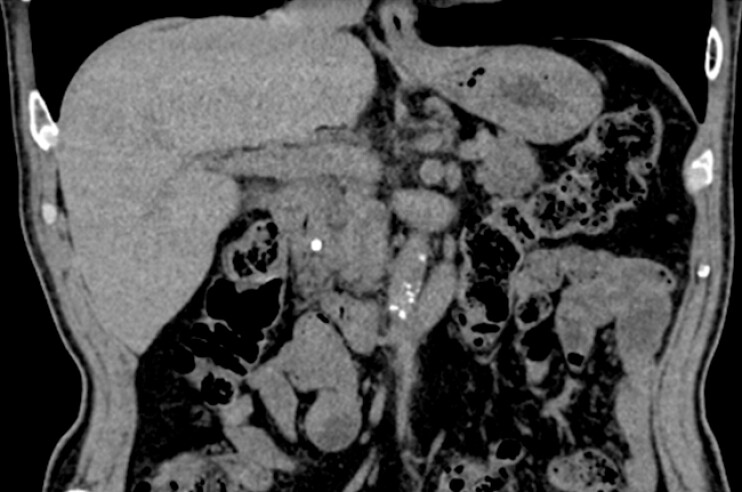
Abdominal computed tomography revealed a 5-mm stone in the duct of Santorini with associated pancreatic duct dilation.

A 73-year-old man with a history of acute pancreatitis was diagnosed with a pancreatic duct stone. Computed tomography revealed a 5-mm stone in the Santorini duct with upstream ductal dilation ( Fig. 1 ). Magnetic resonance cholangiopancreatography showed poor visualization of the Wirsung duct, consistent with pancreatic divisum.

**Fig. 2:**
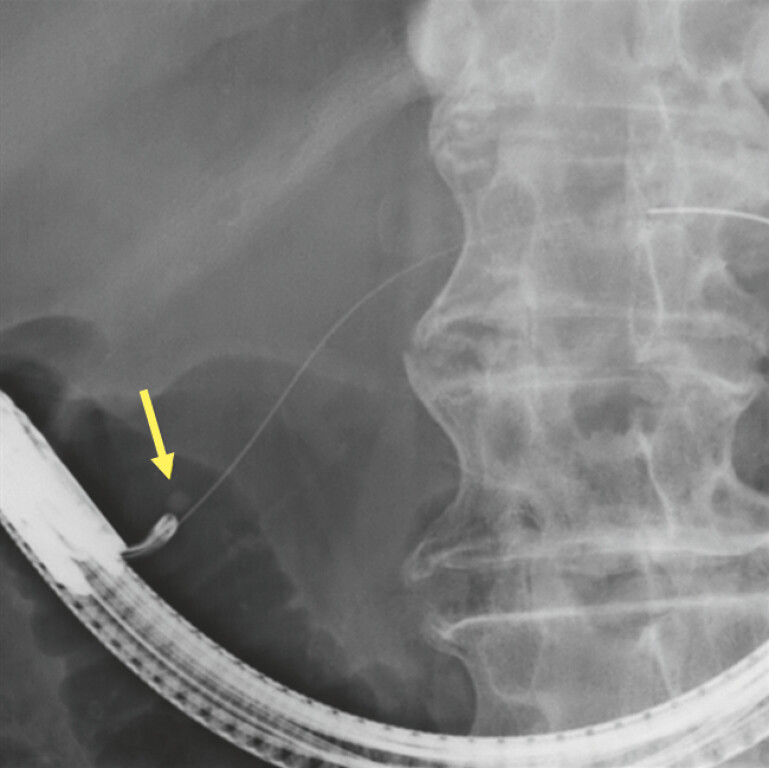
Endoscopic retrograde pancreatography was performed via the minor papilla. A pancreatic stone was in the Santorini duct (arrow).

**Fig. 3:**
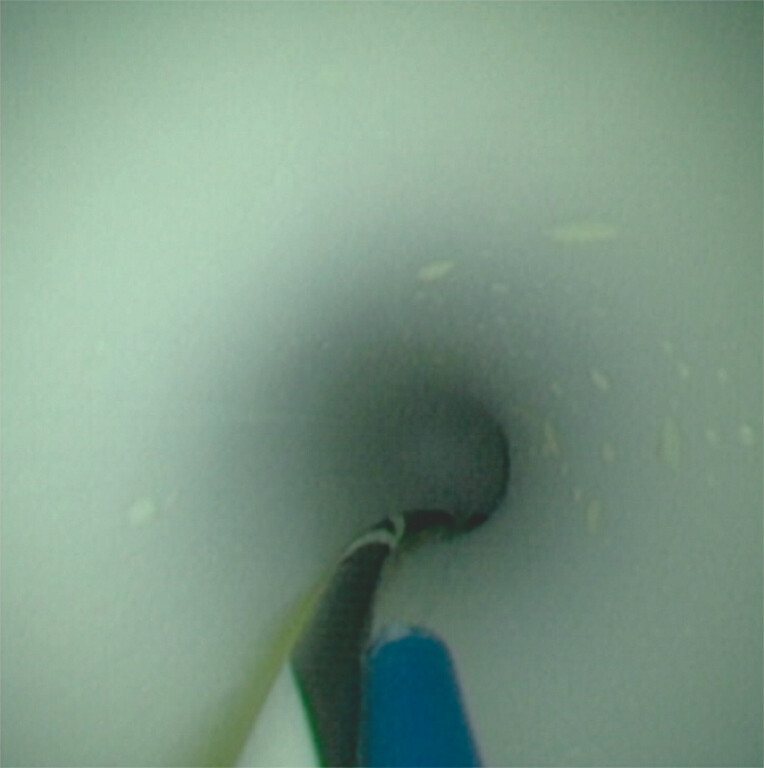
The laser fiber was preloaded in the working channel of the peroral panceratoscope alongside a 0.025-inch guidewire within the duodenoscope.

**Fig. 4:**
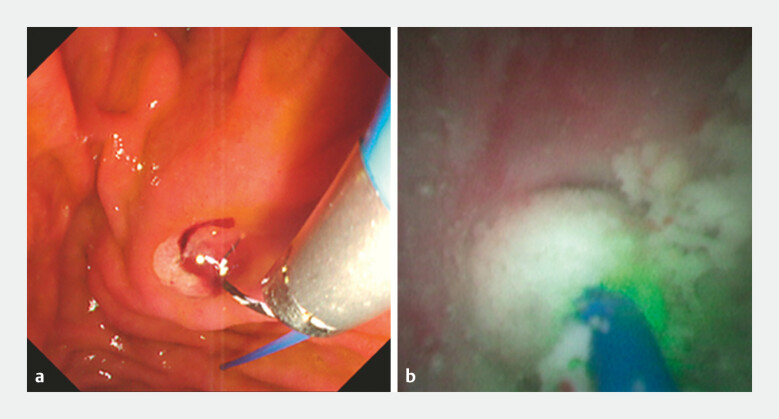
Guidewire-assisted advancement enabled safe pancreatic duct cannulation. **a** Smooth advancement over the curved tip of the duodenoscope and peroral pancreatoscopy. **b** Laser lithotripsy was successfully performed.

Endoscopic retrograde cholangiopancreatography was performed via the minor papilla ( Fig. 2 ). Because the stone could not be extracted using a balloon catheter, holmium:YAG (yttrium-aluminum-garnet) laser lithotripsy (Quanta Litho EVO GI, Quanta System, Milan, Italy) was performed. A 9-Fr cholangiopancreatoscopy (eyeMAX, MICRO-TECH Co., Ltd., Nanjing, China) was advanced over a 0.025-inch guidewire, allowing the clear visualization of the stone. However, laser fiber insertion was unsuccessful because of the sharp angulation of the mother duodenoscope and the POPS tip. To overcome this, the POPS was withdrawn into the duodenoscope, proximal to the duodenoscope’s bending section, and the laser fiber was preloaded in parallel with the guidewire through the working channel of the POPS ( Fig. 3 ). The POPS was then reinserted with the laser fiber positioned at its tip, enabling smooth passage across the angulated segment. Laser lithotripsy was successfully performed, followed by complete stone extraction ( Fig. 4 , Video 1 ). This preloading technique improves laser fiber deliverability in a confined pancreatic duct and may facilitate safer and smoother lithotripsy.

A guidewire-parallel laser fiber preloading technique facilitates reliable laser fiber delivery during peroral pancreatoscopy-guided pancreatic duct stone lithotripsy.Video 1

Endoscopy_UCTN_Code_TTT_1AR_2AL
